# Novel endoluminal parameters for predicting primary loss of response in Crohn’s disease: a multi-center study

**DOI:** 10.1186/s13244-025-02118-y

**Published:** 2025-10-25

**Authors:** Ruchen Yao, Changsheng Cai, Jie Liang, Feng Tian, Xiaocang Cao, Yue Li, Yubei Gu, Qi Feng, Jun Shen, Mimic Inflammatory Bowel Disease Research Group, Feng Tian, Yubei Gu, Qi Feng, Jun Shen, Yu Bai, Zhaolian Bian, Qian Cao, Xiaocang Cao, Kang Chao, Jie Chen, Linlin Chen, Min Chen, Yan Chen, Yuanwen Chen, Cong Dai, Xueli Ding, Juan Du, Jianhua Duan, Yihong Fan, Baisui Feng, Jing Feng, Caiping Gao, Hongliang Gao, Quanyi Gao, Wensong Ge, Sizhen Gu, Hong Guo, Lianyi Guo, Hui Hou, Lihong Jia, Qi Jiang, Jinghua Kuai, Jin Li, Qin Li, Wenli Li, Wenqin Li, Yongyu Li, Jie Liang, Wangdi Liao, Jiang Liu, Haimei Lv, Wen Lv, Ren Mao, Qian Ren, Zhen Sun, Jiawen Wang, Lei Yang, Hongjie Zhang, Jianmin Zhao, Changyu Zhou, Lanxiang Zhu

**Affiliations:** 1 Renji Hospital, School of Medicine, Shanghai Jiao Tong University; Division of Gastroenterology and Hepatology, Key Laboratory of Gastroenterology and Hepatology, Ministry of Health, Inflammatory Bowel Disease Research Center; Shanghai Institute of Digestive Disease, Shanghai, China; 2 NHC Key Laboratory of Digestive Diseases, Shanghai, China; 3 State Key Laboratory of Holistic Integrative Management of Gastrointestinal Cancers and National Clinical Research Center for Digestive Diseases, Xijing Hospital of Digestive Diseases, Fourth Military Medical University, Xi’an, China; 4 Department of Gastroenterology, Shengjing Hospital of China Medical University, Shenyang, China; 5 Department of Gastroenterology and Hepatology, Tianjin Medical University General Hospital, Tianjin Medical University, Tianjin, China; 6 Department of Gastroenterology, Peking Union Medical College Hospital, Chinese Academy of Medical Sciences & Peking Union Medical College, Beijing, China; 7 Department of Gastroenterology, Ruijin Hospital Affiliated to Shanghai Jiao Tong University School of Medicine, Shanghai, China; 8 Department of Radiology, Renji Hospital, School of Medicine, Shanghai Jiao Tong University, Shanghai, China; 9 Department of Gastroenterology, Changhai Hospital, Naval Medical University, Shanghai, China; 10 Nantong Institute of Liver Disease, Nantong Third People’s Hospital, Nantong University, Nantong, China; 11 Department of Gastroenterology, Sir Run Run Shaw Hospital, Zhejiang University School of Medicine, Hangzhou, China; 12 Department of Gastroenterology and Hepatology, Tianjin Medical University General Hospital, Tianjin Institute of Digestive Disease, Tianjin Key Laboratory of Digestive Diseases, Tianjin, China; 13 Department of Gastroenterology, The Sixth Affiliated Hospital, Sun Yat-Sen University, Guangzhou, China; 14 Department of Gastroenterology, Northern Jiangsu People’s Hospital, Yangzhou, China; 15 Fourth Department of the Digestive Disease Center, Suining Central Hospital, Shanghai, China; 16 Department of Gastroenterology, Zhongnan Hospital of Wuhan University School of Medicine, Wuhan, China; 17 Center for Inflammatory Bowel Diseases, Department of Gastroenterology, The Second Affiliated Hospital, Zhejiang University School of Medicine, Hangzhou, China; 18 Department of Gastroenterology, Huadong Hospital Affiliated to Fudan University, Shanghai, China; 19 Department of Gastroenterology, First Hospital of China Medical University, Shenyang City, China; 20 Department of Gastroenterology, The Affiliated Hospital of Qingdao University, Qingdao, China; 21 Department of Gastroenterology, The First Affiliated Hospital, Zhejiang University School of Medicine, Hangzhou, China; 22 Department of Gastroenterology, Shaoxing Hospital of Traditional Chinese Medicine, Shaoxing, China; 23 Department of Gastroenterology, The First Affiliated Hospital of Zhejiang Chinese Medical University, Key Laboratory for Pathophysiological Research on Digestive System Diseases of Zhejiang Province, Hangzhou, China; 24 Department of Gastroenterology, Second Affiliated Hospital of Zhengzhou University, Zhengzhou, China; 25 Shanghai Key Laboratory of Gut Microecology and Associated Major Diseases Research, Digestive Disease Research and Clinical Translation Center, Department of Gastroenterology, Shanghai Ninth People’s Hospital, School of Medicine, Shanghai Jiao Tong University, Shanghai, China; 26 Department of Gastroenterology, Sichuan Provincial People’s Hospital, University of Electronic Science and Technology of China, Chengdu, China; 27 The Second Department of Gastroenterology, The First Affiliated Hospital of Xinjiang Medical University, Urumqi, China; 28 Department of Gastroenterology, Zhongshan Hospital of Xiamen University, School of Medicine, Xiamen University, Xiamen, China; 29 Department of Gastroenterology, Xinhua Hospital, Shanghai Jiaotong University School of Medicine, Shanghai, China; 30 Traditional Chinese Medicine Department, Shuguang Hospital Affiliated to Shanghai University of Traditional Chinese Medicine, Shanghai, China; 31 Department of Gastroenterology, Chongqing General Hospital, University of Chinese Academy of Sciences, Chongqing, China; 32 Department of Gastroenterology, The First Affiliated Hospital of Jinzhou Medical University, Jinzhou, China; 33 Department of Gastroenterology, The Fifth Affiliated Hospital of Xinjiang Medical University, Urumqi, China; 34 Department of Gastroenterology, Jilin Central General Hospital, Jilin, China; 35 Department of Gastroenterology, The First Affiliated Hospital of Ningbo University, Ningbo, China; 36 Department of Gastroenterology, Qilu Hospital of Shandong University (Qingdao), Qingdao, China; 37 Gastroenterology, The Eighth Affiliated Hospital of Sun Yat-sen University, Guangzhou, China; 38 Department of Gastroenterology, Kunshan Second People’s Hospital, Suzhou, China; 39 Department of Gastroenterology, Qingdao Municipal Hospital, Qingdao, China; 40 Department of Gastroenterology, The First Hospital of Jilin University, Changchun, China; 41 Department of Gastroenterology, Affiliated Hospital of Yanbian University, Yanji, China; 42 State Key Laboratory of Cancer Biology, National Clinical Research Center for Digestive Diseases and Xijing Hospital of Digestive Diseases, Fourth Military Medical University, Xi’an, China; 43 Department of Gastroenterology, The First Affiliated Hospital, Nanchang University, Nanchang, China; 44 Huzhou Central Hospital, Affiliated Central Hospital Huzhou University, Huzhou, China; 45 Department of Gastroenterology, Red Cross Hospital of Yulin City, Yulin, China; 46 Department of Gastroenterology, Affiliated Hang Zhou First People’s Hospital, Zhejiang University School of Medicine, Hangzhou, China; 47 Department of Gastroenterology, First Affiliated Hospital of Sun Yat-sen University, Guangzhou, China; 48 The First Hospital of Lanzhou University, Lanzhou, China; 49 Department of Gastroenterology, Jilin People’s Hospital, Jilin City, Jilin, China; 50 Department of Anorectal Surgery, Longhua Hospital, Shanghai University of Traditional Chinese Medicine, Shanghai, China; 51 Department of Endoscopy, China-Japan Union Hospital of Jilin University, Changchun, Jilin, China; 52 Department of Gastroenterology, The First Affiliated Hospital of Nanjing Medical University, Nanjing, China; 53 Department of Gastroenterology, The First Affiliated Hospital of Soochow University, Suzhou, China

**Keywords:** Primary loss of response, Ustekinumab, Crohn’s disease, Endoluminal parameters

## Abstract

**Objectives:**

Approximately 30% of patients with Crohn’s disease (CD) experience primary loss of response (PLR) to ustekinumab. However, studies integrating imaging parameters to predict PLR remain limited. This study aimed to quantify endoluminal and intestinal wall parameters using computed tomography enterography (CTE) and assess their predictive value for PLR to ustekinumab.

**Materials and methods:**

This multicenter study analyzed 466 intestinal segments from 161 patients with CD between March 2020 and May 2024. A national survey identified 10 CTE parameters for evaluating disease activity and predicting PLR. Logistic regression models were used to assess predictive performance in a validation cohort.

**Results:**

Ten CTE parameters related to lesion characterization—including length, luminal narrowing, and bowel wall thickness—were defined, with newly introduced metrics, including length, area, effective luminal diameter (EffLD), mean bowel wall thickness, and stenosis. A total of 352 baseline and 114 follow-up segments from 161 patients across six centers were analyzed to assess changes following ustekinumab treatment. Paired analysis across all patients showed significant improvements in eight parameters (*p* < 0.001); in contrast, unpaired comparisons between PLR and non-PLR groups revealed significant differences in six parameters (*p* < 0.001), with greater improvements in the non-PLR group. EffLD emerged as an independent predictor of ustekinumab response, with an AUC of 0.858 and an accuracy of 0.780 in the validation cohort.

**Conclusions:**

Novel endoluminal parameters, particularly EffLD, provide a detailed characterization of intestinal lesions in inflammatory bowel disease and exhibit strong predictive value for PLR in ustekinumab-treated patients with CD.

**Critical relevance statement:**

This study first applied cardiovascular imaging software to quantify endoluminal CT enterography parameters, establishing effective luminal diameter as a novel, clinically applicable predictor of ustekinumab response in Crohn’s disease.

**Key Points:**

- Cardiovascular imaging software can facilitate image analysis in Crohn’s disease.
- Endoluminal CT enterography parameters predict primary loss of response in Crohn’s disease.
- Effective luminal diameter independently predicts ustekinumab response.

**Graphical Abstract:**

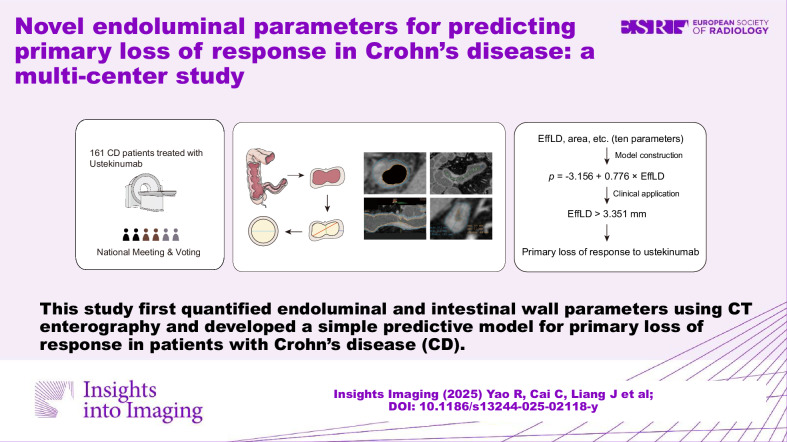

## Introduction

Crohn’s disease (CD) is a chronic autoimmune disorder of the digestive tract and a form of inflammatory bowel disease (IBD) [1]. Although incurable, treatment aims to maintain remission and prevent complications. Biologic therapies, including infliximab and ustekinumab, target inflammatory pathways to modulate immune responses and are considered advanced treatment options for CD [2]. Although ustekinumab effectively reduces intestinal wall thickening and relieves stenosis [3], approximately 30% of patients experience primary loss of response (PLR), resulting in inadequate symptom relief [4]. Persistent disease activity worsens prognosis, increasing complications and the need for surgical intervention.

Currently, no reliable method exists to predict PLR, and therapy selection often depends on clinical experience and “sequential therapy.” Multi-omics-based predictive models show promise but are limited by high costs and complex analyses. Laboratory markers such as C-reactive protein, hemoglobin, anti-drug antibodies, and cytokine levels are widely available, but have inconsistent predictive value [5–9]. Mucosal cytokine levels offer a more direct assessment of local inflammation, but require invasive endoscopy, which is unsuitable for strictured regions [10].

Computed tomography enterography (CTE) is an essential imaging tool in CD, providing a comprehensive assessment of lesions across all bowel segments, including strictured areas inaccessible to endoscopy [11]. However, existing radiomics models for predicting PLR face challenges in interpretability and clinical applicability [12–15]. Although CTE evaluates intestinal wall thickness, luminal stenosis, lesion extent, and complications, the irregularity of intestinal lesions complicates quantification, making assessments highly dependent on radiologist interpretation [16]. To address this, we drew inspiration from cardiovascular imaging software, which shares similarities with intestinal lumen analysis.

Cardiovascular imaging software is well-developed for assessing luminal and wall structures. Given the parallels in luminal morphology, optimal distension, and contrast enhancement, adapting these techniques for intestinal analysis should be feasible.

This study applied cardiovascular imaging techniques to quantify intestinal CTE parameters, introducing a novel parameter, EffLD, to characterize intestinal lesions and predict primary loss of response (PLR) to ustekinumab in Crohn’s disease (CD).

## Materials and methods

### Sample size

This study was approved by the Institutional Review Board of Renji Hospital, Shanghai Jiao Tong University (KY2019-039). Sample size was determined based on the small shrinkage of predictor effects proposed by Riley et al [17]. A minimum sample size of 136 lesioned segments was required in the training cohort depending on the following conditions and assumption: outcome proportion, 0.40; shrinkage factor: 0.90; number of candidate predictors: 10; alternative hypothesis of the area under the receiver operating characteristic (ROC) curve (AUC): 0.80 (null hypothesis of the AUC: 0.50). Therefore, a total sample size of 352 lesions (252 in the training cohort and 100 in the validation cohort; PLR: 147) was deemed sufficient for this study.

### Study population

**Fig. 1:**
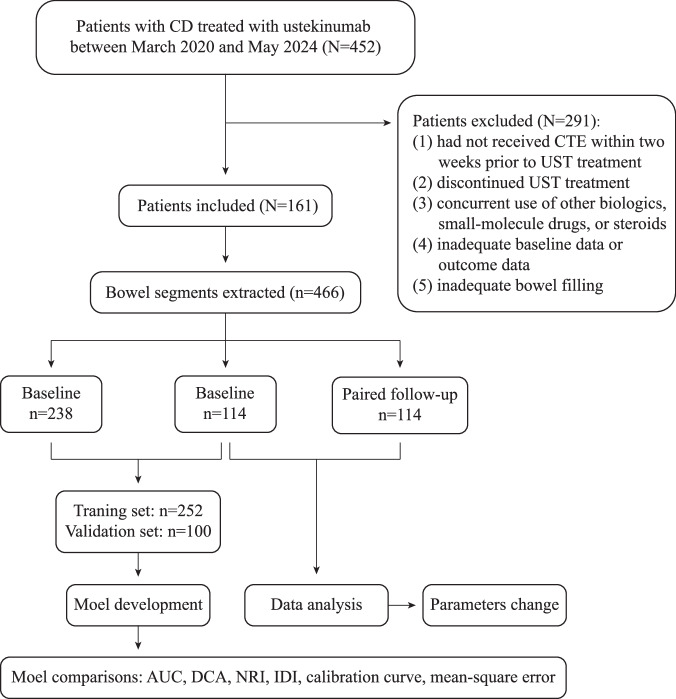
Flowchart of the study design

This multicenter retrospective study analyzed 466 segments from 161 patients with CD in six hospitals between March 2020 and May 2024. The baseline cohort included 106 patients with 352 lesions from six hospitals, and the paired follow-up cohort included 55 patients with 114 lesions from Renji Hospital, with CTE scans obtained at 6 months after ustekinumab initiation. CD was diagnosed according to the European Crohn’s and Colitis Organization criteria [18]. The inclusion criteria were as follows: (1) a confirmed diagnosis of CD; (2) received ustekinumab (Janssen Pharmaceuticals) in an induction dose of 6 mg/kg and a maintenance dose of 90 mg every 8 weeks. The exclusion criteria were as follows: (1) patients had not received CTE within two weeks prior to ustekinumab treatment; (2) discontinued ustekinumab treatment; (3) concurrent use of other biologics, small-molecule drugs, or steroids; (4) those with missing baseline clinical data or outcomes; or (5) cases of inadequate bowel filling. Patients with prior abdominal surgery were excluded if lesions were located at anastomosis or adhesion sites. A flowchart of the study design is shown in Fig. 1.

### Outcomes and definitions

PLR was determined by a multidisciplinary team comprising at least gastroenterologists, radiologists, and pathologists. Surgeons were also involved if patients presented a surgical risk or had undergone surgery. The multidisciplinary team integrated clinical indices, imaging, endoscopy, and laboratory data for evaluation. PLR was defined if any of the following criteria were met within 24 weeks of ustekinumab initiation: (1) Harvey–Bradshaw Index (HBI) ≥ 4 or an increase in the HBI to > 3 [19]; (2) Crohn’s Disease Activity Index (CDAI) ≥ 150 or an increase in the CDAI to > 100 [20]; (3) Simple Endoscopic Score for Crohn’s Disease (SES-CD) > 3 or a reduction in the SES-CD to < 50% [21]; (4) Magnetic Resonance Activity Index (MaRIA) ≥ 11 [22]; and (5) a need for therapy modification, including dose escalation, switching to alternative biologics or small-molecule drugs, corticosteroids, combination of immunosuppressive therapy, or CD-related surgery [23].

### CTE examination and parameter measurement

CTE examinations were conducted according to a standardized protocol within 2 weeks prior to ustekinumab treatment among centers. Patients were required to fast for 12 h before the procedure and were administered 1500 mL of polyethylene glycol solution (Heshuang, Wanhe Pharmaceutical; or Fortrans, Ipsen Pharma) in three 500 mL doses for 45, 30, and 15 min prior to the scan. CT scans were taken from the diaphragm to the perineum using 64-detector CT scanners (GE MEDICAL SYSTEMS Revolution GSI; uCT 960+, UNITED IMAGING; SIEMENS SOMATOM Force, Siemens Healthcare) while the patients were positioned supine and instructed to hold their breath for the duration of the scan. Contrast-enhanced imaging was performed following an intravenous injection of 1.5 mL/kg of the contrast agent (Iopamiro, Bracco Sine) at a rate of 3 mL/s. The scanning parameters of the GE MEDICAL SYSTEMS Revolution GSI were as follows: tube current, 5 mAs; voltage, 120 kV; collimation, 40 mm; and pitch, 1.375:1. The scanning details of uCT 960+ were as follows: tube current, 235 mAs; voltage, 120 kV; collimation, 80 mm; and pitch, 0.9937:1. Scanning details of SIEMENS SOMATOM Force were as follows: tube current, 135 mAs; voltage, 100 kV; collimation, 76.8 mm; and pitch, 0.6:1. The images were reconstructed with a thickness of 1.0 mm, with enteric phase images captured 70 s after administering the contrast agent for further analysis.

The parameter measurements were conducted by a radiologist with 20 years of experience (Intraclass Correlation Coefficient > 0.95). CT images of the enteric phase obtained 70 s after contrast agent administration were retrieved for image reconstruction and analysis. All measurements and analyses were completed by the “coronary artery analysis module” on the PHILIPS CT workstation (IntelliSpace Portal 12.1.4) following the official instructions (https://www.documents.philips.com). The entire process included the following key steps: (1) target intestinal lesions with active inflammation were identified on multiplanar reconstruction images (mainly coronal images) in each patient. (2) The center point of the intestinal lumen was manually set from the beginning of the intestinal lesion to its end along the intestinal lumen. The position of the center point in the axial or sagittal image was adjusted to ensure that it was in the center of the intestinal lumen as much as possible. The number of selected points mainly depended on the length or route of the intestinal lesions. The longer or more tortuous the intestinal lesion, the more central are the points required. (3) The intestinal lesion would be “stretched” straightened in the same two-dimensional (2D) plane along the previously set center points. (4) In this reconstructed image, the narrowest point of the intestinal lesion was chosen, and the software automatically generated detailed parameters. (5) A healthy segment near the lesioned segment was analyzed as a control by following the above steps.

### Model construction and comparison

Patients were randomly assigned to training and validation cohorts in a 7:3 ratio [24]. Logistic models were initially developed using the training cohort. Variables with *p* < 0.2 in univariate analysis were retained for the least absolute shrinkage and selection operator (LASSO) with 10-fold cross-validation [25]. Backward stepwise logistic regression was performed, and the final model was selected based on the lowest Akaike information criterion (AIC).

Model performance was assessed in the validation cohort. The ROC and calibration curve were generated to evaluate the predictive accuracy. Decision curve analysis (DCA) was performed to assess the net benefits of clinical applications. The accuracy, specificity, sensitivity, and mean square error were calculated to measure model performance. Improvements were quantified using the integrated discrimination improvement index (IDI) and net reclassification improvement index (NRI).

### Statistical analysis

Data analysis was performed using R, version 4.2.1 (https://www.r-project.org/). The AUCs of different models were compared using DeLong’s method. The accuracy, specificity, and sensitivity were calculated using the thresholds based on the Youden index or the point nearest to (0, 1) in the ROC plot. Numerical data were presented as mean ± standard deviation (for normally distributed data) or median (interquartile range) (for non-normally distributed data). Categorical variables are expressed as numbers (percentages). Baseline data and parameter changes were analyzed using the Student’s *t*-test, Wilcoxon rank-sum test, or Fisher’s exact test, as appropriate. A two-sided *p*-value of < 0.05 was considered statistically significant.

## Results

### A national survey

An online national survey was conducted in 1 week among 121 multidisciplinary physicians, with 95 completing the survey (Method S1). Participant characteristics are detailed in Supplementary Table S1. All proposed metrics were classified as “recommended,” as the combined percentage of “totally agree” and “agree” responses exceeded 80% across all scales. Among the eight candidate indicators, bowel position (68%), stenosis (67%), and mean bowel wall thickness (67%) received the highest “totally agree” votes for association with disease activity. These metrics were also the top predictors of PLR, with stenosis (59%), bowel wall thickness (56%), and bowel position (54%) receiving the most votes. Additionally, four metrics that have been the subject of recent debates were included in the questionnaire, which similarly received a high percentage of approval. The free-text section generated 28 and 23 suggestions for two categories, which were reviewed and classified as either “supplements (potential additional indicators)” or “suggestions” based on the description. The survey results are detailed in Supplementary Table S2.

**Fig. 2:**
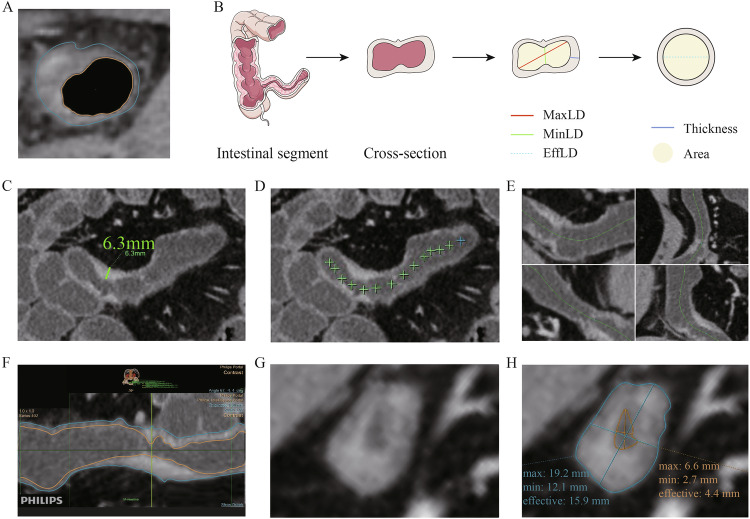
Definition and extraction of parameters. **A** A cross-section of the intestine illustrates the irregular shape of the intestinal lumen. **B** Endoluminal parameters were obtained from the cross-section of a bowel segment. **C** The green line indicates a thickened intestinal wall at a lesion site. **D** The center points of the intestinal lumen were manually set from the beginning to the end of the intestinal lesion along the lumen. **E** The center points were adjusted in axial or sagittal images to ensure precise placement within the lumen. **F** The intestinal lesion was “stretched” and straightened within the same 2D plane along the predefined center points. **G**, **H** In this reconstructed image, the narrowest point of the intestinal lesion was selected, and then the software automatically generated detailed parameters after adjusting the tracing lines. MaxLD, maximum luminal diameter; MinLD, minimum luminal diameter; EffLD, effective luminal diameter

Based on the survey findings, 10 CTE parameters were identified, including nine highly rated parameters and one newly introduced parameter—effective luminal diameter (EffLD). Given the irregular shape of the intestinal lumen (Fig. 2A), experts determined that minimum luminal diameter (MinLD) and maximum luminal diameter (MaxLD) alone were insufficient to fully capture the luminal features. Consequently, EffLD was introduced as an area-derived parameter, calculated using the formula: area = π × (EffLD/2)^2^. Definitions and details are provided in Table 1, with illustrations and extraction examples shown in Fig. 2B–H.

**Table 1:** Definition of parameters

CTE parameters	Unit	Types of data	Definition
Segments	-	Ordinal	Number of lesioned intestinal segments^a^ 1: < 5 2: 5–10 3: ≥ 10
Position	-	Nominal	Position of the lesioned segment^b^ 1: Intestine 2: Colon or rectum
Length	mm	Numerical	Length of a continuous segment of lesioned intestine
Area	mm^2^	Numerical	Cross-sectional area of the lesioned intestinal lumen
Minimum luminal diameter (MinLD)	mm	Numerical	Minimum diameter of the lesioned intestinal lumen
Maximum luminal diameter (MaxLD)	mm	Numerical	Maximum diameter of the lesioned intestinal lumen
Effective luminal diameter (EffLD)	mm	Numerical	Area-derived effective diameter of the lesioned intestinal lumen^c^
Thickness	mm	Numerical	Mean bowel wall thickness (BWT) of the lesioned intestine
Relative thickness	-	Numerical	BWT of the lesioned intestine/BWT of the health control^d^
Stenosis	-	Numerical data between 0 and 1	(Area of the health control-Area of the lesioned intestine)/Area of the health control

*BWT* bowel wall thickness, *MaxLD* maximum luminal diameter, *MinLD* minimum luminal diameter, *EffLD* effective luminal diameter

^a^ Lesioned intestinal segments: Segments with wall thickening (> 3 mm) or stricture (defined as lumen narrowing with proximal small bowel dilation [16])

^b^ Upper gastrointestinal involvement is not included due to its small proportion

^c^ Area = π × (EffLD/2)^2^

^d^ Health control: Normal segment of the colon and/or the intestine adjacent to the site of the lesion(s)

### Patient characteristics

A total of 161 patients were included in the study, with baseline clinical characteristics summarized in Supplementary Table S3. To study the CTE parameters change, paired baseline and follow-up data were obtained for 114 intestinal segments (45 PLR and 69 non-PLR) from 55 patients. To develop the prediction model, 352 baseline lesioned bowel segments from 106 patients were extracted, with 252 segments (103 PLR and 149 non-PLR) assigned to the training set and 100 segments (44 PLR and 56 non-PLR) included in the validation set.

### Change in CTE parameters after treatment with ustekinumab

CTE scan and parameter measurement were performed as described in Supplementary Method S1. Significant improvements in imaging parameters were observed at the first CTE follow-up (week 24) after ustekinumab administration (*p* < 0.001 for all parameters; Table 2). Bowel wall thickness and stenosis were reduced following ustekinumab treatment, along with a decrease in the length of affected segments. Furthermore, three parameters of lumen diameter (MinLD, MaxLD, and EffLD) and the lumen area were increased. These improvements in CTE parameters suggest that ustekinumab has a positive effect on intestinal lesions, with a reduction in the extent of lesions and inflammatory activity.

**Table 2:** Changes in CTE parameters after ustekinumab treatment

	Baseline (*N* = 114)	Endpoint (*N* = 114)	*p*-value
Length (mm)	70.00 (42.00, 126.00)	54.50 (26.00, 84.00)	< 0.001
MinLD (mm)	1.90 (1.40, 3.20)	3.90 (2.00, 7.20)	< 0.001
MaxLD (mm)	3.70 (2.20, 6.40)	7.85 (4.30, 12.90)	< 0.001
EffLD (mm)	2.65 (1.80, 4.40)	5.60 (3.00, 10.70)	< 0.001
Area (mm^2^)	5.65 (2.70, 15.80)	24.85 (7.10, 89.50)	< 0.001
Thickness (mm)	6.93 (6.00, 8.10)	4.78 (3.40, 6.60)	< 0.001
Relative thickness	4.74 (3.96, 6.32)	3.38 (2.48, 4.28)	< 0.001
Stenosis	0.98 (0.92, 0.99)	0.90 (0.59, 0.96)	< 0.001

The position and segments were not included, as no changes were noted between baseline and follow-up measurements

*MaxLD* maximum luminal diameter, *MinLD* minimum luminal diameter, *EffLD* effective luminal diameter

**Fig. 3:**
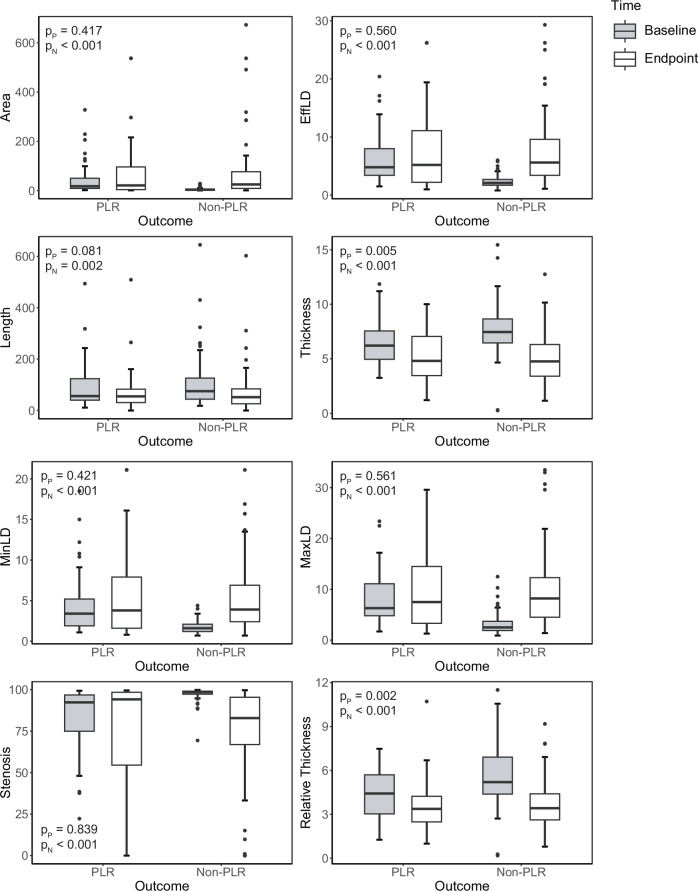
Changes in CTE parameters between PLR and non-PLR groups. The non-PLR group showed greater improvements in CTE parameters compared to the PLR group. MinLD, minimum luminal diameter; MaxLD, maximum luminal diameter; EffLD, effective luminal diameter; p_N_, *p*-value of the non-response group; p_R_, *p*-value of the response group

Next, we compared changes in CTE parameters between the PLR and non-PLR groups (Table S4). In the non-PLR group, eight parameters (i.e., all except position and segments) showed a significant improvement; in contrast, in the PLR group, only thickness and relative thickness changed significantly, suggesting limited imaging improvement (Fig. 3). Further analysis of differences in parameter changes between the two groups (Supplementary Table S5) revealed statistically significant differences in all parameters except segment length, with greater improvements in the non-PLR group. Notably, in the PLR group, MinLD and MaxLD exhibited opposing trends, reinforcing the importance of EffLD as a key parameter for capturing luminal changes.

### Construction of models based on CTE parameters

Based on survey findings and clinical data analysis, 10 CTE parameters were considered for model development. In the training cohort, nine parameters (*p* < 0.2; with length excluded, *p* = 0.215; Table 3) underwent 10-fold cross-validation using LASSO (Supplementary Fig. S1). This process identified five variables with nonzero coefficients: segments, position, EffLD, area, and relative thickness. Backward stepwise regression, based on the AIC, further refined the model to include area and EffLD, resulting in three models: EffLD, area, and their combination. The model coefficients and intercepts are listed in Supplementary Table S6.

**Table 3:** Univariate analysis of CTE parameters

Parameters	OR	95% CI	*p*-value
Segments			
< 5	1.00		
5–10	0.61	0.36–1.03	0.066
> 10	0.92	0.35–2.40	0.862
Position			
Intestine	1.00		
Colon or rectum	2.39	1.27–4.49	0.007
Length (mm)	1.00	1.00–1.00	0.215
MinLD (mm)	2.70	2.00–3.63	< 0.001
MaxLD (mm)	1.41	1.26–1.57	< 0.001
EffLD (mm)	2.17	1.74–2.72	< 0.001
Area (mm^2^)	1.11	1.07–1.15	< 0.001
Thickness (mm)	0.80	0.69–0.92	0.002
Relative thickness	0.69	0.58–0.82	< 0.001
Stenosis	0.00	0.00–0.00	< 0.001

*OR* odds ratio, *CI* confidence interval, *MaxLD* maximum luminal diameter, *MinLD* minimum luminal diameter, *EffLD* effective luminal diameter

### EffLD is an independent CTE predictor of the PLR

**Fig. 4:**
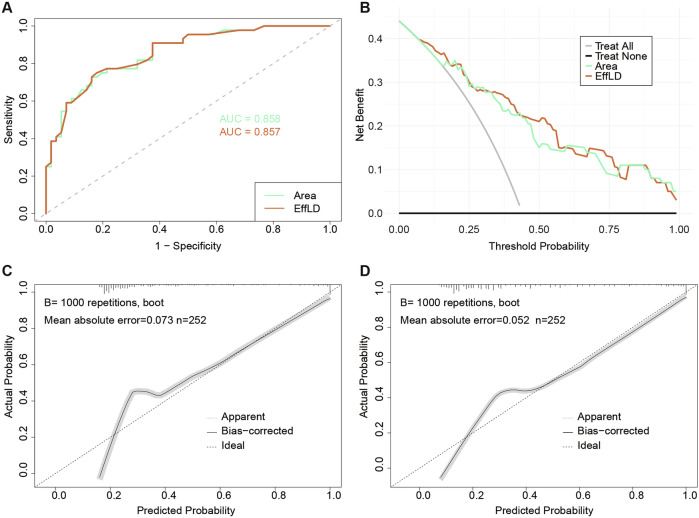
Performance evaluation of the EffLD and Area models. **A** ROC curves for two models in the validation sets. **B** Decision curve analysis (DCA) curves for two models in the validation sets. Calibration plot for (**C**) Area model and (**D**) the EffLD model in the validation sets. EffLD, effective luminal diameter; ROC, receiver operating characteristic curve

Area and EffLD are mathematically related (area = π × (EffLD/2)^2^), which precludes their simultaneous inclusion in the model despite the weak multicollinearity (variance inflation factor: 4.488). In the training cohort, the EffLD model had a lower AIC than the area model (233.04 vs. 245.87). We then evaluated the model performance in the validation cohort by considering the EffLD and area as independent variables (Table 4). The AUC and calibration curves for the two models are nearly identical, with ROC values of 0.858 and 0.857 (*p* = 0.919), respectively. However, DCA showed a slightly better performance for the EffLD model than for the area model (Fig. 4). The EffLD model also demonstrated higher accuracy and specificity as well as a lower mean square error, although its sensitivity was lower. Furthermore, IDI analysis showed a 4.78% improvement in predictive performance for the EffLD model compared to the area model (95% CI: 0.0283–0.0673; *p* < 0.001); in contrast, NRI was not significant (3.73%; 95% CI: −0.0661 to 0.1407; *p* = 0.479). Despite comparable diagnostic performance between the two parameters, EffLD was selected as the independent endoluminal predictor of PLR due to its clinical interpretability and slightly superior performance in prediction. The final prediction model was: *p* = −3.156 + 0.776 × EffLD. Accordingly, when the EffLD at the narrowest point of the diseased bowel segment exceeds 3.351 mm, the patient is predicted to exhibit PLR to ustekinumab.

**Table 4:** Comparison between EffLD model and Area model

Predictor	AUC	Accuracy	Sensitivity	Specificity	Mean square error
Training cohort					
EffLD model	0.866	0.758	0.845	0.698	7.747
Area model	0.863	0.762	0.874	0.685	33.322
Validation cohort					
EffLD model	0.857	0.790	0.750	0.821	3.983
Area model	0.858	0.780	0.773	0.786	9.191

*AUC* area under the ROC curve, *EffLD* effective luminal diameter

## Discussion

This study identified CTE parameters that are easily interpretable via a national survey and characterized them to predict the PLR to ustekinumab in patients with CD. Leveraging the tubular structural similarity between the intestine and blood vessels, we applied cardiovascular imaging software to analyze CTE images. This method enables noninvasive, rapid prediction of treatment response, potentially guiding therapeutic decisions before drug initiation and reducing the likelihood of ineffective treatment. To address previous limitations in measuring the length of the curved intestinal tract, we developed a method that “stretches” the intestinal tract across different imaging sequences, aligning it to a consistent 2D plane [26]. However, our analysis revealed no significant differences in segment length between the PLR and non-PLR groups, and length did not predict PLR. This may be due to the discontinuous nature of CD lesions, where a single segment does not fully represent disease involvement. Therefore, the inclusion of patients with contiguous lesions or those diagnosed with ulcerative colitis may improve prediction accuracy. Previous studies have used MinLD, MaxLD, and lesion length from CTE and magnetic resonance enterography (MRE) to predict surgical risk in patients with CD [26]. However, these parameters were excluded from our model, as MinLD did not contribute; in contrast, EffLD emerged as an independent predictor.

Our findings align with prior studies showing that ustekinumab alleviates bowel wall thickening and stenosis [3]. In CD, intestinal thickening and luminal narrowing result from inflammation-driven wall thickening. Mechanistically, ustekinumab antagonizes interleukin (IL)–12 and IL-23, attenuating T-cell-mediated inflammation [27] and inhibiting Th1/Th17 pathways, which reduces fibrosis formation [28, 29]. This dual action suggests potential benefits for both inflammatory and fibrotic stenosis in CD. However, existing methods struggle to quantify stenosis-related variations. Although ultrasound-based indicators (for example, luminal diameter and area) are established for CD detection, ultrasound remains operator-dependent and difficult to standardize [30, 31].

Bowel wall thickness is widely considered the most critical indicator of disease activity. However, prior studies calculated thickness as the mean of four cross-sectional measurements, introducing error due to CD’s eccentric lesions [3, 32]. In this study, we implemented a method to more accurately capture average thickness across multiple cross-sections. Interestingly, although bowel thickness was prioritized in the voting process and showed significant changes between PLR and non-PLR groups, it was excluded from the final model. This suggests that bowel wall thickness primarily reflects inflammation rather than ustekinumab efficacy. Previous research indicates that ultrasound-measured bowel wall thickness at visit 1 (weeks 14–26 post-treatment) predicts transmural healing at visit 2 (weeks 44–56 post-treatment) in patients treated with infliximab. However, baseline bowel wall thickness did not predict later healing, nor did visit 1 thickness predict mucosal healing at visit 2 [32]. These findings suggest that the predictive value of bowel wall thickness depends on treatment duration and drug type. Although a previous study found that ustekinumab reduced bowel wall thickness in patients without PLR, our study indicated that ustekinumab improves intestinal parameters regardless of clinical response [3]. Furthermore, greater improvements in CTE parameters in patients without PLR highlight the potential of endoluminal imaging in treatment monitoring and failure prediction. EffLD, an area-driven metric commonly used in cardiovascular imaging [33, 34], has not been widely applied in IBD due to the irregular shape of the bowel lumen, which is affected by uneven lesions. Unlike blood vessels, the variability in CD lesion distribution creates a highly irregular lumen, making EffLD particularly relevant.

This study has some limitations. The sample size restricted our ability to track bowel parameter changes throughout treatment and centers. The relatively small cohort also precluded external validation of the EffLD threshold in a larger, independent dataset. Furthermore, the clinical significance of EffLD—particularly its relationship to intestinal strictures—requires further investigation. Additionally, patients who previously underwent CTE may opt for MRE, a radiation-free alternative, complicating longitudinal comparisons. Physical contraction of the colon can also artificially increase bowel wall thickness, decrease luminal diameter, and shorten segment length. To account for this, colonoscopic images were reviewed to distinguish between contractions and lesions when necessary. However, as a static imaging modality, CTE struggles to differentiate between contracted and inflammatory lesions, a challenge also presents in MRE. This underscores the importance of integrating multiple assessment methods, including endoscopy and ultrasound. Additionally, factors beyond the parameters analyzed—such as complications (for example, fistulas and abscesses) and mesenteric fat—may influence inflammation and treatment response [35–37]. Further research should incorporate these factors.

Expanding cross-platform parameters is essential. In the national survey, panel members proposed additional parameters linked to disease activity and prognosis. Notably, integrating MRE and ultrasound metrics was the most frequently suggested approach. Each modality has unique advantages: ultrasound excels in assessing blood flow and detecting complications like strictures, fistulas, and abscesses [38]; in contrast, MRE provides superior characterization of perianal disease and may correlate T2-weighted signal intensity with inflammatory activity [39]. Although both CTE and MRE are recommended as first-line imaging modalities for CD, most patients undergo only one for diagnosis. Consequently, most patients typically undergo only one of these imaging tests during the evaluation [40]. The parameters developed in this study are straightforward, less susceptible to platform-specific variability, and hold potential for validation across multiple imaging modalities.

In conclusion, this study provides a precise quantification of intestinal parameters, including length, bowel wall thickness, and endoluminal parameters, before and after ustekinumab treatment in patients with CD. Our findings suggest that EffLD may serve as a valuable biomarker for optimizing CD treatment.

## Supplementary information

ELECTRONIC SUPPLEMENTARY MATERIAL

## Data Availability

Data are available from the authors upon reasonable request.

## Abbreviations

AIC: Akaike information criterion
AUC: Area under the ROC curve
CD: Crohn’s disease
CTE: Computed tomography enterography
DCA: Decision curve analysis
EffLD: Effective luminal diameter
IBD: Inflammatory bowel disease
IDI: Integrated discrimination improvement index
LASSO: Least absolute shrinkage and selection operator
MaxLD: Maximum luminal diameter
MinLD: Minimum luminal diameter
MRE: Magnetic resonance enterography
NRI: Net reclassification improvement index
PLR: Primary loss of response
ROC: Receiver operating characteristic
SES-CD: Simple Endoscopic Score for Crohn’s Disease
