# Supplementary material for: Novel endoluminal parameters for predicting primary loss of response in Crohn’s disease: a multi-center study
Source: Insights Imaging. 2025 Oct 25;16:228. doi: 10.1186/s13244-025-02118-y (PMC12553649; doi:10.1186/s13244-025-02118-y)
Supplement: Supplementary file 1 — ELECTRONIC SUPPLEMENTARY MATERIAL [file 13244_2025_2118_MOESM1_ESM.pdf]

Novel endoluminal parameters for predicting primary loss of response in Crohn's disease: A multi-center study.

ELECTRONIC SUPPLEMENTARY MATERIAL

Supplementary file

Method S1. A national survey and parameter selection
Table S1. Characteristics of participants in the survey
Table S2. Results of the national survey
Table S3. Baseline characteristics of CD patients treated with ustekinumab
Table S4. Changes in CTE parameters in two groups after ustekinumab treatment
Table S5. Differential changes in CTE parameters between PLR and non-PLR groups
Table S6. Development of three models
Figure S1. CTE parameters selection using LASSO with ten-fold cross-validation

Method S1. A national survey and parameter selection

A national survey was conducted to define the parameters [1]. Seven core members from six hospitals within the Mimic Inflammatory Bowel Disease Research Group participated. Following offline discussions, a comprehensive questionnaire was developed using *WJX.cn* (Changsha, Hunan, China).

The questionnaire, distributed anonymously by a team member, was sent to the IBD multidisciplinary team (MDT) leaders at 48 hospitals, each of whom invited up to five experts to participate. Responses were rated on a five-point Likert scale: “totally agree,” “agree,” “uncertain,” “disagree,” and “totally disagree.” Parameters were categorized based on voting percentages: (1) **Strongly recommended**: 80% “totally agree” votes. (2) **Recommended**: 80% combined “totally agree” and “agree” votes. (3) **Suggested**: >80% combined “totally agree,” “agree,” and “uncertain” votes. Parameters that did not meet inclusion thresholds were excluded. Group discussions were held before and after voting to finalize parameters selection.

Table S1: Characteristics of participants in the survey.

	No. of participants
**Specialty**	
Gastroenterology	69 (72.63%)
Gastrointestinal surgery	17 (17.90%)
Radiology	7 (7.37%)
Pathology	2 (2.11%)
**Grade**	
Consultant/attending/equivalent	77 (81.05%)
Registrar/resident/equivalent	18 (18.95%)
**Hospital level**	
Regional Center for Inflammatory Bowel Disease[a]	62 (65.26%)
Non-Regional Center for Inflammatory Bowel Disease	33 (34.74%)

[a] The IBD Quality Care Evaluation Center (IBDQCC) certifies IBD units to establish a network of high-quality care for IBD in China. As of October 2024, 97 hospitals in mainland China have been certified as Regional Centers for Inflammatory Bowel Disease. Lists are available at http://ibdqcc.com/.

Table S2. Results of the national survey

	Related to disease activity (%)					Able to predict loss of response (%)				
	**A**	**B**	**C**	**D**	**E**	**A**	**B**	**C**	**D**	**E**
Number of lesioned intestinal segments	52	41	5	0	2	45	42	8	3	1
Position of the lesioned segment: Intestine, colon, or upper gastrointestinal involvement	68	22	7	1	1	54	41	4	1	0
Length of a continuous segment of lesioned intestine	47	42	11	0	0	49	45	4	1	0
Cross-sectional area of the lesioned intestinal segment	59	27	13	1	0	46	41	11	2	0
Minimum diameter of the lesioned intestine lumen	61	26	11	1	1	46	37	12	4	1
Maximum diameter of the lesioned intestine lumen	57	28	12	2	1	44	38	13	4	1
Mean bowel wall thickness of the lesioned intestine	67	28	3	1	0	56	36	8	0	0
Stenosis: (Area of the health control-Area of the lesioned intestine)/Area of the health control	67	25	5	2	0	59	31	9	1	0
Active or inactive inflammation	71	21	7	0	1	67	25	5	1	1
Presence of fistula	67	26	4	2	0	69	26	3	1	0
Abundance of blood supply	64	31	5	0	0	58	37	5	0	0
Mesenteric fat proliferation	65	32	2	1	0	55	41	3	1	0
**Supplements**	**Related to disease activity**				**n**	**Able to predict loss of response**				**n**
	Ultrasound or MRE data				8	Ultrasound or MRE data				3
	CT value				3	Number of mesenteric lymph nodes				3
	Radiomics				2	Intestinal fibrosis				2
	Fistula				2	Course of disease				1
	Abdominal abscess				2					
**Suggestions**	Diameter and area should be assessed with adequate dilation of the intestinal lumen.<br>Simple parameters are insufficient for accurate prediction; radiomics should be considered.<br>Prediction models should be developed based on indicators of intestinal inflammation.<br>Severe imaging features before treatment may be linked to poor outcomes, though supporting data is limited.<br>Minimum and maximum diameters alone are inadequate for characterizing the lesioned lumen due to the irregular shape of lumen.									

A: Totally agree; B: Agree; C: Uncertain; D: Disagree; E: Totally disagree.

Table S3. Baseline characteristics of CD patients treated with ustekinumab

	Response (N = 90)	Loss of response (N = 71)	Total (N = 161)	*p*
**Sex (male/female)**	55/35	39/32	94/67	0.529
**BMI, kg/m^2**				0.133
<18.5	26 (28.89%)	15 (21.13%)	41 (25.47%)	
18.5-24.9	59 (65.56%)	46 (64.79%)	105 (65.22%)	
≥24.9	5 (5.56%)	10 (14.08%)	15 (9.32%)	
**Age, year**				0.818
18-40	61 (67.78%)	46 (64.79%)	107 (66.46%)	
≥40	29 (32.22%)	25 (35.21%)	54 (33.54%)	
**Course, median (IQR),**	2.50 (0.00, 6.00)	4.00 (1.00, 8.00)	3.00 (0.00, 7.00)	0.264
**Biologics**				0.036
Bio-naïve	61 (67.78%)	32 (45.07%)	93 (57.76%)	
Infliximab	19 (21.11%)	29 (40.85%)	48 (29.81%)	
Adalimumab	1 (1.11%)	3 (4.23%)	4 (2.48%)	
Vedolizumab	4 (4.44%)	3 (4.23%)	7 (4.35%)	
Two biologics	5 (5.56%)	4 (5.63%)	9 (5.59%)	
**Surgery (yes/no)**	37/53	37/34	74/87	0.218
**Location**				0.960
L1	38 (42.22%)	29 (40.85%)	67 (41.61%)	
L2	7 (7.78%)	5 (7.04%)	12 (7.45%)	
L3	45 (50.00%)	37 (52.11%)	82 (50.93%)	
**Upper gastrointestinal**	1/89	1/70	2/159	>0.99
**Behavior**				0.966
B1	35 (38.89%)	27 (38.03%)	62 (38.51%)	
B2	46 (51.11%)	36 (50.70%)	82 (50.93%)	
B3	9 (10.00%)	8 (11.27%)	17 (10.56%)	
**Perianal disease (yes/no)**	73/17	58/13	131/30	>0.99
**ESR, mm/L**[a]				0.747
≤15 or ≤20	44 (48.89%)	32 (45.07%)	76 (47.20%)	
>15 or >20	46 (51.11%)	39 (54.93%)	85 (52.80%)	
**CRP, mg/L**				0.138
≤8	56 (62.22%)	35 (49.30%)	91 (56.52%)	
>8	34 (37.78%)	36 (50.70%)	70 (43.48%)	
**WBC, median (IQR),**	5.95 (4.70, 6.97)	6.04 (5.07, 7.68)	5.96 (4.86, 7.23)	0.358
**Hb, g/L**[b]				0.563
<130 or <115	42 (46.67%)	29 (40.85%)	71 (44.10%)	
130-175 or 115-150	48 (53.33%)	42 (59.15%)	90 (55.90%)	
**HCT, %**[c]				0.816
<40 or <35	34 (37.78%)	29 (40.85%)	63 (39.13%)	
≥40 or≥35	56 (62.22%)	42 (59.15%)	98 (60.87%)	
**PLT, median (IQR), 10^9/L**	309.50 (253.00, 358.00)	288.00 (241.50, 368.00)	300.00 (247.00, 362.00)	0.522
**ALB, median (IQR), g/L**	40.75 (37.90, 44.10)	40.30 (36.20, 42.80)	40.50 (37.50, 43.60)	0.153

Location L1: ileal; L2: colonic; L3: ileocolonic; Behavior B1: non-stricturing and non-penetrating; B2: stricturing; B3: penetrating.

[a] The threshold value for ESR is 15 mm/L in males and 20 mm/L in females.

[b] The threshold value for Hb is 130-175 g/L in males and 115-150 g/L in females.

[c] The threshold value for HCT is 40% in males and 35% in females.

Table S4. Changes in CTE parameters in two groups after ustekinumab treatment

	PLR group			Non-PLR group		
	**Baseline** (N=45)	**Endpoint** (N=45)	***p* value**	**Baseline** (N=69)	**Endpoint** (N=69)	***p* value**
**Length**	56.00 (40.00, 124.00)	55.00 (31.00, 83.00)	0.081	75.00 (44.00, 126.00)	52.00 (26.00, 84.00)	0.002
**MinLD**	3.40 (1.90, 5.20)	3.80 (1.60, 7.90)	0.421	1.60 (1.20, 2.10)	3.90 (2.40, 6.90)	<0.001
**MaxLD**	6.30 (4.80, 11.10)	7.50 (3.30, 14.50)	0.561	2.50 (1.90, 3.70)	8.20 (4.50, 12.30)	<0.001
**EffLD**	4.80 (3.40, 8.00)	5.20 (2.20, 11.10)	0.560	2.10 (1.70, 2.70)	5.60 (3.40, 9.60)	<0.001
**Area**	18.00 (8.80, 50.30)	21.00 (4.00, 96.20)	0.417	3.60 (2.10, 5.90)	25.00 (8.90, 76.70)	<0.001
**Thickness**	6.20 (4.95, 7.55)	4.80 (3.45, 7.05)	0.005	7.45 (6.45, 8.65)	4.75 (3.40, 6.30)	<0.001
**Relative Thickness**	4.42 (3.03, 5.70)	3.37 (2.48, 4.23)	0.002	5.20 (4.38, 6.91)	3.42 (2.62, 4.40)	<0.001
**Stenosis**	0.92 (0.75, 0.97)	0.94 (0.54, 0.98)	0.839	0.98 (0.97, 0.99)	0.83 (0.67, 0.95)	<0.001

MaxLD: maximum luminal diameter; MinLD: minimum luminal diameter; EffLD: effective luminal diameter.

Table S5. Differential changes in CTE parameters between PLR and non-PLR groups

	**Non-PLR** (N=69)	**PLR** (N=45)	***p* value**
**Length** (mm)	-17.00 (-67.00, 7.00)	-9.00 (-51.00, 10.00)	0.345
**MinLD** (mm)	2.10 (0.60, 5.00)	0.10 (-1.70, 3.40)	<0.001
**MaxLD** (mm)	4.30 (1.40, 9.20)	-0.10 (-2.70, 5.00)	<0.001
**EffLD** (mm)	3.20 (1.10, 7.00)	-0.30 (-10.20, 54.70)	<0.001
**Area** (mm^2)	20.00 (4.10, 74.20)	-0.30 (-10.20, 54.70)	<0.001
**Thickness** (mm)	-2.25 (-4.25, -1.15)	-0.90 (-2.20, 0.55)	<0.001
**Relative Thickness**	-1.96 (-3.12, -0.91)	-0.93 (-1.82, 0.29)	0.001
**Stenosis**	-0.13 (-0.30, -0.03)	0.01 (-0.29, 0.06)	<0.001

For the columns of "Non-PLR" and "PLR", negative values indicate a decrease in these parameters from baseline to follow-up, while positive values indicate an increase. PLR: loss of response. MaxLD: maximum luminal diameter; MinLD: minimum luminal diameter; EffLD: effective luminal diameter.

Table S6. Development of three models

Model 1: EffLD + Area

Variables	β	OR (95% CI)	*p* value
(Intercept)	-3.729	0.024 (0.009, 0.067)	<0.001
EffLD	1.089	2.973 (2.018, 4.380)	<0.001
Area	-0.044	0.957 (0.924, 0.991)	0.013

Model 2: EffLD

Variables	β	OR (95% CI)	*p* value
(Intercept)	-3.156	0.043 (0.019, 0.094)	<0.001
Diameter	0.776	2.173 (1.736, 2.720)	<0.001

Model 3: Area

Variables	β	OR (95% CI)	*p* value
(Intercept)	-1.712	0.181 (0.114, 0.285)	<0.001
Area	0.106	1.112 (1.072, 1.153)	<0.001

OR: odds ratio, CI: confidence interval

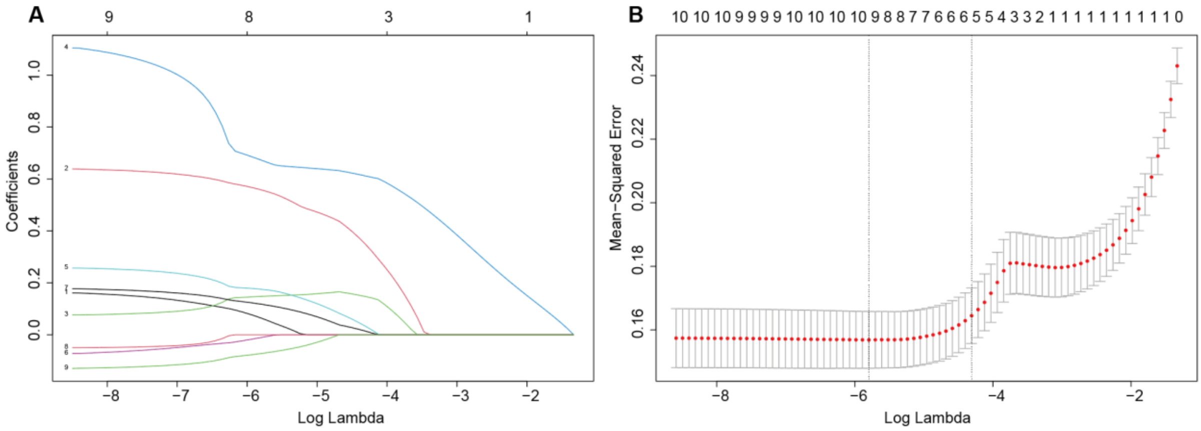

Figure S1. CTE parameters selection using LASSO with ten-fold cross-validation. **A**. LASSO coefficient profiles of CTE parameters. A coefficient profile plot was drawn versus the selected log(λ) value, and 4 variables with nonzero coefficients were generated. **B**. Optimal λ selection in the LASSO model using ten-fold cross-validation. Dotted vertical lines indicate the optimal λ values based on the 1 standard error of the minimum criteria. A λ value of 0.0134 with was selected, resulting in 5 variables with nonzero coefficients.

Reference

1. Nasa P, Jain R, Juneja D. Delphi methodology in healthcare research: How to decide its appropriateness. *World Journal of Methodology*. **11**, 116-129 (2021).
